# Perspectives of Stakeholders of Equitable Access to Community Naloxone Programs: A Literature Review

**DOI:** 10.7759/cureus.21461

**Published:** 2022-01-20

**Authors:** Lucas Martignetti, Winnie Sun

**Affiliations:** 1 Faculty of Health Sciences, Ontario Tech University, Oshawa, CAN

**Keywords:** overdose, overdose prevention, opioid epidemic, health inequity, opioids, harm reduction, naloxone

## Abstract

The purpose of this review is to examine the existing literature about facilitators and barriers influencing equitable access to naloxone programs by individuals who use opioids. A total of 49 published articles were examined, which generated four overarching themes:(1) Stigma as a barrier to access; (2) Lack of a wide range of stakeholder perspectives; (3) Need for a comprehensive understanding of factors affecting equitable access to naloxone programs; (4) Facilitators to increase the access of community naloxone programs. Our review highlighted the importance of advocacy in practice, education, administration, and policy to address the health inequities that exist in naloxone distribution programs. Advocacy activities involve the need for health care professionals to engage in social justice practice through evidence-based informed research about the facts of opioid use; challenging the stigma toward victim-blaming against naloxone users; as well as promoting program development and health policy to bring about equitable access to naloxone programs by marginalized and socially disadvantaged populations.

## Introduction and background

Opioid-related harms are currently at an all-time high across Canada, the United States, and around the world. In 2020 alone, preliminary data show 6,200 opioid-related deaths in Canada and over 69,000 opioid-related deaths in the United States [1-2]. Opioids are a family of drugs that work to relieve pain through the inhibition of the release of neurotransmitters that transmit pain signals throughout the body. Much of the media coverage of opioid use refers to illicit drugs, including heroin and fentanyl. This does not factor in the contributions of opioid drugs medically prescribed for pain management such as morphine, oxycodone, and codeine. Extensive research has been done into identifying the root cause of the opioid crisis as it exists across North America. Increased opioid harms have been attributed to both increased prescription of opioids such as oxycodone as well as illicit and diverted drugs such as heroin, fentanyl, and carfentanil [3-5]. While all socioeconomic groups are being affected by the opioid crisis, disparities can be seen in different populations. For example, individuals living in low-income communities in Ontario, Canada, have experienced harm at a substantially higher rate than those who lived in high-income communities [6]. Similar findings are seen in the United States [7-8]. As well, a First Nations individual living in Canada is five times more likely to experience an opioid-related overdose and three times more likely to die from an overdose than a non-First Nations individual [9].

Opioid overdose is treated initially through the delivery of naloxone, also known by its various brand names, most popularly Narcan, through either needle injection or the application of nasal spray [10-11]. This is followed by professional medical attention (from first responders, emergency nurses, and physicians) with an initial focus on supporting the patient’s airway, breathing, and circulation, as well as administering additional naloxone as necessary [12-13]. The literature highlighted that naloxone administration should not be limited to healthcare professionals but rather, it could be delivered by anyone who has received proper education from healthcare providers regarding its safe administration [14-16]. Naloxone works to reverse an opioid overdose by acting as an antagonist to opioid receptors [17]. Without accessible naloxone, overdoses cannot be reversed until emergency services arrive, which may delay successful treatment outcomes. At this time, the individual may experience several symptoms, one of the most dangerous being respiratory depression [18]. Hypoxia resulting from respiratory depression can potentially lead to brain damage, paralysis, or death while the likelihood of these adverse events increases with the delay of naloxone administration as a result of inequitable access [18-20].

Across North America, there exist a number of programs allowing individuals to receive naloxone kits in a number of settings, often without cost or a prescription. These are often referred to as either Take Home Naloxone (THN) or Opioid Education and Naloxone Distribution (OEND) programs. In Canada, there exist programs funded by provincial governments accessible by the public, including the Ontario Naloxone Program and the British Columbia Take Home Naloxone Program [21]. In the United States, programs tend to be focused around smaller geographical areas, such as municipalities, as seen with programs like the Drug Overdose Prevention and Education (DOPE) Project in San Francisco, California [22].

Similar naloxone programs around the world have significantly increased the odds of recovery after an overdose [14]. However, the number of accidental opiate-related deaths remains high, despite programs implemented to deliver the anti-overdose drug naloxone to people who use opioids, as well as their friends and families [1]. To better understand how to improve the effectiveness of these programs, existing research on barriers and/or facilitators for naloxone access generally focuses on quantitative studies of a single group of stakeholders. An example of this includes a study using an online questionnaire given to Canadian physicians to determine their perceived barriers to naloxone access [23]. Some qualitative studies have been implemented with the stakeholders of naloxone distribution programs, including community pharmacists. Additional studies have been conducted in other countries, including Australia [24], and in settings not available to the public such as correctional facilities [25]. A gap exists in the literature where a wide range of important stakeholders have not been given a voice to share their lived experiences with the barriers and facilitators that exist in the current naloxone distribution programs.

The purpose of this review is to examine the existing literature about the perspectives of stakeholders from community naloxone programs in regards to facilitators and barriers to equitable access and will address the following two objectives: (1) To identify the socio-cultural factors influencing equitable access as perceived by those who participate or would participate in community naloxone programs; (2) to explore knowledge gaps in the existing literature to identify areas where equitable access to community naloxone programs can be improved.

## Review

Methods

Research Method

The research method used here was the process model as applied by Engert et al. (2016) [26], which was adapted from the work of Mayring (2014) [27]. The literature review consists of four steps. The first is a structured material collection, the second is a descriptive review of the material collected, the third step is the categorization of material, and the fourth and final step is the evaluation of material collected.

Inclusion and Exclusion Criteria

The search strategy for the review included journal articles, research papers, and gray literature published until June 2021. Searches were conducted on the topic and revealed little research has been done in the field of community naloxone programs. Searches were not limited by year to allow for a more comprehensive search about the study topic. Keywords used included “substance use” or “substance abuse” or “opioid use” or “opioid addiction” or “overdose” to encompass literature that examined the use of opioids. The keywords “naloxone” or “narcan” were used to limit the search to this specific medication and its most common brand name, rather than any other medications used in relation to opioid use. Other keywords included “barriers” or “challenges” and “facilitators” or “enablers” as well as “perspectives” or “attitudes” or “views” or “perceptions” or “perceived”, in order to explore literature documenting the lived experiences of individuals utilizing the naloxone programs and their perceptions related to what facilitated and hindered their access. The criteria for article inclusion was that the literature must focus on naloxone use for opioid use and addiction and overdose reversal. The included articles must have a primary focus on the concept of access to naloxone treatment. The criteria for exclusion included studies that focused on the clinical or therapeutic use of opioids and opioid maintenance therapies. Studies focused on naloxone use for opioid addiction and overdose reversal in institutional or correctional facilities were excluded due to the distinct policies and protocols in these naloxone distribution programs, as well as the inability of the general public to access these programs.

Search Strategy and Analysis

**Figure 1:**
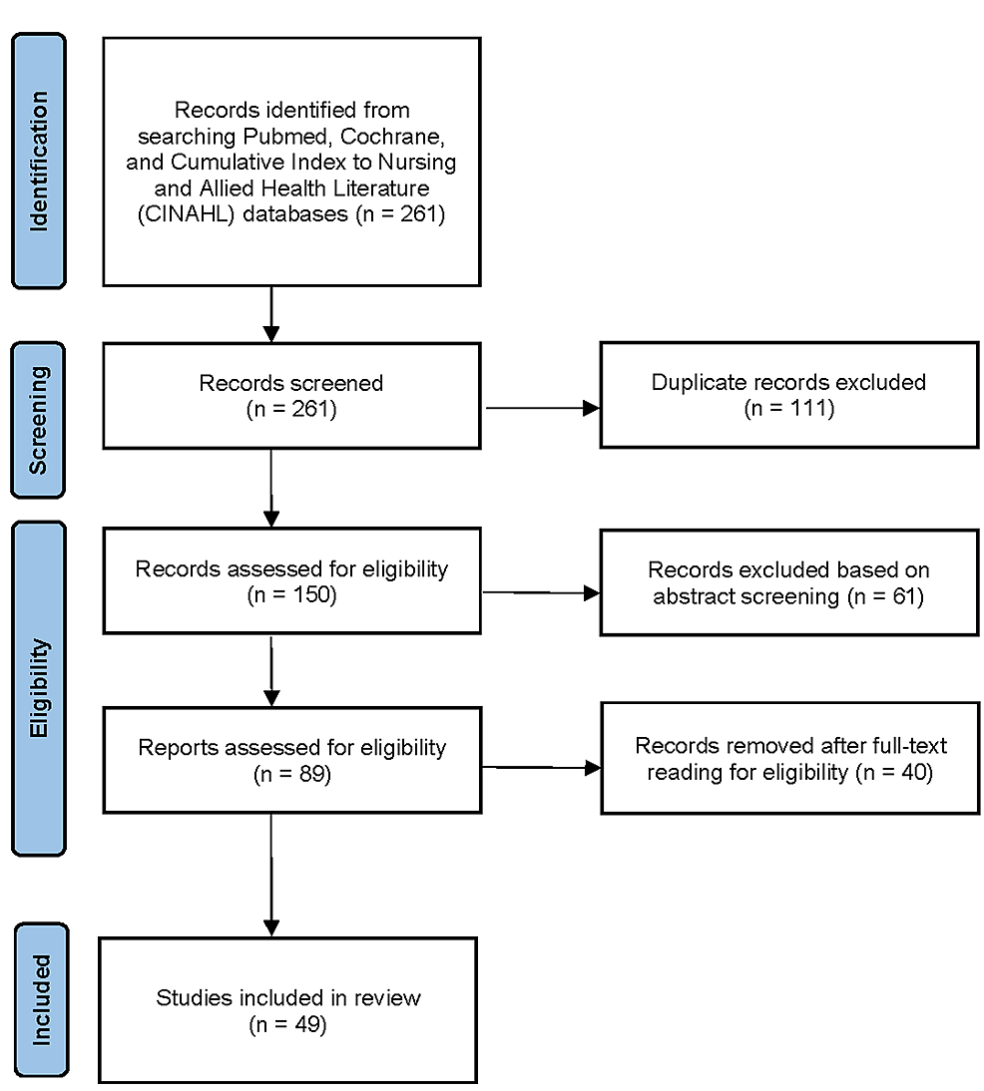
PRISMA flow diagram PRISMA, Preferred Reporting Items for Systematic Reviews and Meta-Analyses; n, number of studies

To conduct the literature review, searches were completed using the library databases of the Ontario Tech University, including the PubMed, Cochrane, and Cumulative Index to Nursing and Allied Health Literature (CINAHL) databases. The search strategy across all databases was consistent, using the same Boolean search string. Full-text articles from peer-reviewed journals were selected for inclusion, along with editorials, letters, books, protocols, and gray literature. All search results were retrieved for further review, which yielded a final retrieval of 136 publications in total. The abstracts of all retrieved literature were reviewed by each author and only relevant publications that addressed the objectives were kept for data extraction. This was followed by an assessment of all the relevant full-text articles. The reference lists of articles were examined for additional relevant literature for inclusion. This process can be seen in Figure 1. A total of 49 published articles were found to be relevant to our objectives, a summary of which can be found in Table 1.

**Table 1:** Summary of articles included in the discussion. BCTHN, British Columbia take-home naloxone, THN, take-home naloxone; PWID, people who inject drugs; PWUD, people who use drugs; OEND, overdose education and naloxone distribution; OOPP, opioid overdose prevention program

Author (date)	Study purpose	Study design	Summary of key findings relevant to this review
Antoniou et al. [28] (2021)	To assess the experiences of individuals who had accessed naloxone from a community pharmacy, relating these experiences to the risk environments and broader naloxone discourses.	Qualitative in-depth interviews were completed with 37 participants.	Participants experienced stigma when accessing naloxone, feared legal repercussions as well as tapering of opioid doses. Participants preferred less judgmental naloxone distribution sites.
Bachhuber et al. [29] (2015)	To determine the types of messaging that can increase public support for naloxone distribution policies in the United States.	A web-based randomized survey was distributed to an address-based random sampling of households, with 1 598 respondents.	Providing facts about opioid use and/or providing sympathetic narratives to respondents led to higher support for naloxone distribution and laws protecting those administering naloxone.
Bakhireva et al. [30] (2018)	To identify what barriers and facilitators may exist to dispensing intranasal naloxone by pharmacists in New Mexico.	Questionnaires were distributed to pharmacists, with 390 respondents.	Pharmacists surveyed responded that: naloxone administration may lead to continued or riskier opioid use, that distributing naloxone from their pharmacy may bring in clientele that would negatively affect their business, that the cost of naloxone to clients may prohibit access, and that there may be insufficient time to properly train and educate clients on naloxone administration.
Bartlett et al. [31] (2011)	To examine understandings of risk factors related to overdose from a local perspective, assessing ongoing barriers to overdose response, and soliciting input from clients of a harm reduction program in Geiju, China.	30 qualitative interviews were completed in total, comprised of 15 individuals who had overdoses reversed through naloxone injections, and 15 individuals who called the hotline of the harm reduction program in response to witnessing an overdose.	Participants pointed to local changes in heroin use such as the aging of the opioid using population as well as drug mixing practices that increase the risk of overdose. Avoidance of emergency service providers was seen as a result of concerns that medical professionals may be unwilling to treat PWUD, that their confidentiality may not be protected, and that high financial costs associated with treatment function as a barrier to its access.
Beletsky et al. [32] (2007)	To assess the willingness and knowledge of physicians to prescribe naloxone.	A questionnaire was mailed and faxed to a sample of physician members of the American Medical Association, with 588 respondents.	Respondents believe that naloxone administration may lead to continued or riskier opioid use.
Bessen et al. [33] (2019)	To better understand the experiences and opinions of emergency responders and opioid users relating to naloxone use and distribution in New Hampshire.	112 semi-structured interviews were conducted with opioid users and emergency responders.	Respondents believed that access to naloxone may enable increased or riskier use of opioids. As well, they believe that naloxone does not address underlying issues of addiction and that it may prevent people who use opioids from visiting an emergency department following an overdose. Perceived barriers to naloxone access include prohibitively high costs to clients, legal concerns, lack of knowledge of how to administer naloxone, stigma towards people who use opioids, painful withdrawal after being administered naloxone.
Boeri and Lamonica [34] (2021)	To better understand opioid use in suburban communities.	105 interviews and short surveys were conducted on people who use opioids residing in suburban areas in Georgia, Massachusetts, and Connecticut.	Participants’ ability to access naloxone varied greatly in different locations.
Bowles and Lankenau [35] (2019)	Exploring the diffusion process of opioid overdose prevention programs (OOPPs) among persons who inject drugs (PWID).	30 qualitative interviews were completed among PWID in Philadelphia to identify key themes.	Barriers to participating in OOPPs included the belief that training was either too time-consuming or unnecessary. Participants stated hesitance to administer naloxone as the recipient may respond aggressively.
Bounthavong et al. [36] (2019)	To identify the perceived barriers and facilitators to dispensing naloxone among providers after the implementation of a national academic detailing program.	Semi-structured interviews were conducted with 11 participants, consisting of physicians, clinical psychiatric pharmacists, and nurse practitioners.	A barrier identified was the lack of support for homeless program users. Facilitators identified were: creating lists of program users, repeat visits, and face-to-face and one-on-one video conferencing.
Childs et al. [37] (2021)	To identify and better understand the challenges and strategies to expand harm reduction services and engage communities in accepting harm reduction perspectives and services.	Qualitative interviews were conducted with 22 professionals working with people who use drugs.	Respondents found that general harm reduction programs, including those that distribute naloxone, face stigma from multiple sources including law enforcement and the general community.
Chronister et al. [38] (2018)	To evaluate the Overdose Prevention and Emergency Naloxone Project (OPENP) THN program in Australia.	Training for the OPENP was given with baseline, post-training, and in-depth interviews six months following training.	Among trainees, there was fear of legal repercussions for calling emergency services when responding to an opioid overdose.
Deonarine et al. [39] (2016)	To examine perspectives related to the British Columbia take-home naloxone (BCTHN) program held by participants, as well as perspectives of law enforcement relating to naloxone administration by police officers.	2 focus groups were conducted with individuals who had received BCTHN training, 2 in-depth interviews were conducted with experienced BCTHN naloxone administrators, and 2 in-depth interviews were conducted with law enforcement officials.	Respondents believe that naloxone administrators may be reluctant to contact emergency services due to legal repercussions.
Donovan et al. [40] (2020)	To understand perceptions of leaders for pharmacy organizations regarding ways for pharmacies and their staff to optimize naloxone dispensing.	In-depth interviews were conducted with 12 pharmacy leaders.	Facilitators identified include: decreasing stigma towards addiction and opioid use, decreasing hesitancy to offer naloxone to patients, coordination of efforts across pharmacies including training.
Dwyer et al. [24] (2016)	To identify perspectives and experiences of service providers relating to THN programs in Victoria, Australia.	15 in-depth interviews were conducted with service providers who are either involved with THN programs or work in a capacity where they are in contact with people who use opioids.	Service providers interviewed held perspectives that: those administering naloxone will be endangered due to aggressive symptoms from the naloxone recipient experiencing withdrawal, there may be insufficient time to provide clients with naloxone training, and that a lack of knowledge of legal liability related to naloxone prescription and administration can deter both.
Edwards et al. [41] (2017)	To assess attitudes held by pharmacists toward the THN program in Alberta and to identify how better to support pharmacists engagement in the program	A questionnaire was e-mailed to clinical pharmacists registered with the Alberta College of Pharmacists, with 470 responses.	Respondents believed that: stigma from the community acted as a barrier to clients, that naloxone kits are not user-friendly, that naloxone programs were poorly advertised, that there may be insufficient time to properly train clients on naloxone administration, that they may face legal repercussions when dispensing or administrating of naloxone, and that distributing naloxone through their pharmacy may invite clientele that would negatively affect their business.
Freeman et al. [42] (2017)	To assess the willingness of pharmacists in Kentucky to dispense naloxone.	A questionnaire was e-mailed to all licensed Kentucky pharmacists, with 1282 respondents.	Respondents stated that there may be insufficient time to properly train clients on naloxone administration, that naloxone administration may lead to continued or riskier opioid use, and that distributing naloxone through their pharmacy may invite clientele that would negatively affect their business
Gatewood et al. [43] (2016)	To determine barriers to naloxone prescription to third-party contacts of people who use opioids (including family members, friends, bystanders) by medical providers.	10 in-depth interviews and three focus group discussions were completed, collecting data from 30 individuals, including academic physicians and medical students.	Barriers included: the belief that providing naloxone would lead to continued or riskier opioid use in the future, costs of naloxone being prohibitive to clients, and lack of knowledge of legal liability related to naloxone administration.
Gilbert et al. [44] (2020)	To better understand the knowledge and attitudes of pharmacists working in rural community pharmacies regarding naloxone.	All 364 rural community pharmacies in Georgia were contacted by phone and asked about naloxone distribution using a “secret shopper” methodology.	Pharmacists can serve as gatekeepers, can act as barriers or facilitators to access based on knowledge of naloxone, perceptions of those seeking naloxone.
Green et al. [45] (2013)	To explore interventions to reduce deaths from opioid overdoses.	143 in-depth interviews were conducted in total, with the study focusing analysis on 24 interviews with health providers working in emergency departments.	Medical providers interviewed believed that providing naloxone may lead to continued or riskier opioid use.
Green et al. [46] (2017)	To explore pharmacists’, caregivers’, and naloxone consumers’ attitudes towards pharmacy-based THN programs and opioid safety in Massachusetts and Rhode Island.	8 focus groups were conducted.	Respondents believed that clients of THN programs would fear discrimination from pharmacists and not participate in the program. There were suggestions to have a system where clients can indirectly communicate with pharmacists that they need naloxone rather than identify themselves as part of the stigmatized group of people who use drugs.
Green et al. [47] (2020)	To examine experiences obtaining naloxone from community pharmacies, and reactions of stakeholders to communication tools and outreach materials promoting the use of naloxone.	8 focus groups were conducted.	Respondents noted stigma when obtaining naloxone from a pharmacy. Respondents also noted fear of legal repercussions or not being prescribed pain medication in the future.
Gunn et al. [48] (2018)	To assess all existing literature that examines naloxone distribution from the ED.	Systematic review.	Respondents believe the costs of naloxone are potentially prohibitive to clients.
Haggerty and Gatewood [49] (2018)	To explore awareness of opioid overdose, as well as to identify perceptions of naloxone and benefits and barriers to naloxone dispensing and administration by community pharmacies in Virginia.	A paper-based questionnaire was distributed to adults in public locations, with 139 respondents.	Respondents believe that naloxone administration may lead to continued or riskier opioid use
Hammett et al. [50] (2014)	To assess law and policy documents, as well as the knowledge, attitudes, and practices of key stakeholders of those involved in programs supporting PWID in six countries (United States, Russia, Vietnam, China, Canada, and Mexico).	Systematic review	Studies reviewed found that clients of THN programs would fear discrimination from health providers and not participate in the program, that distributing naloxone through their pharmacy may invite clientele that would negatively affect their business, and that the costs of naloxone are potentially prohibitive to clients
Holland et al. [51] (2019)	To explore perceptions of THN in ED settings held by physicians and pharmacists,	25 in-depth interviews were conducted with ED pharmacists and physicians,	Interviewed physicians and pharmacists held perspectives that stigma may function as a barrier to accessing THN, that naloxone administration may lead to continued or riskier opioid use, and that there may be insufficient time to properly train clients on naloxone administration
Lai et al. [52] (2021)	To evaluate if drug use patterns change in response to naloxone availability and to explore individuals’ relationship with naloxone.	A pilot study was conducted with semi-structured interviews conducted with 10 participants.	Naloxone kits were considered easy to obtain as they were made available at no cost and training was provided. Participants suggested naloxone be provided to all people leaving needle exchange or treatment programs, and that mobile outreach programs be implemented.
Lewis et al. [15] (2016)	To evaluate the OEND program of the Baltimore Student Harm Reduction Coalition.	Training for the OEND program was given with pretest and posttest surveys, and follow-up telephone surveys 8 to 12 months later. 113 individuals completed the pretest and posttest surveys. 35 individuals completed the follow-up telephone survey.	The training allowed individuals to become more confident with naloxone administration, and less fear of trouble from law enforcement for doing so.
Mahon et al. [53] (2018)	To explore incoming pharmacy students’ baseline knowledge of and attitudes toward harm reduction to create a curriculum that produces pharmacists able to reduce the harm caused by the opioid crisis.	Questionnaires were distributed to first-year pharmacy students, with 167 respondents.	Many students lacked the knowledge to effectively respond to an opioid overdose and were unfamiliar with naloxone. Some students were unwilling to respond in any way other than calling an ambulance. Many students used stigmatizing language towards people who use opioids and felt that naloxone would enable continued or riskier use of opioids.
Martino et al. [54] (2020)	To identify barriers to prescribing naloxone in an OEND program established in an academic health system.	Mixed methods were used, with a questionnaire completed by 72 respondents, made up of physicians and pharmacists, 34 of which participated in a telephone interview.	Facilitators identified include an increased social normalization and acceptability of naloxone use. Barriers identified include the belief that naloxone will encourage increased opioid use, stigma towards addiction and opioid use, and a lack of naloxone training and education for prescribers
McAuley et al. [55] (2018)	To grow the evidence base of THN by examining the lived experience of THN use.	8 qualitative interviews were completed among individuals who had used naloxone from a THN program to reverse an overdose.	The use of naloxone to reverse an overdose is both an emotionally and practically complex experience. Witnessing withdrawal following an overdose reversal was sometimes distressing, but not seen as a barrier to naloxone use. All participants were willing to apply naloxone.
Meyerson et al. [56] (2020)	To explore the feasibility of establishing a harm reduction intervention program in pharmacies, including the distribution of naloxone.	Surveys were completed by 303 Indiana managing pharmacists.	Barriers to naloxone provision include time constraints and the cost to naloxone recipients.
Mitchell et al. [16] (2017)	To address existing knowledge gaps in literature exploring the experiences of young adults with THN programs, and to identify areas for improvement solicited from participants of Inner City Youth (ICY) Program in Vancouver, British Columbia.	2 focus groups and 5 in-depth interviews were conducted with ICY program participants.	Respondents stated that naloxone kits should be placed in common areas of low-income housing units.
Muzyk et al. [57] (2019)	To examine pharmacists’ attitudes towards naloxone and medications used in the treatment of opioid use disorder.	A systematic review was conducted.	The literature found in the review found some pharmacists were not comfortable providing naloxone education to patients, often as a result of a perceived lack of training on the subject. As well, pharmacists reported not being comfortable dispensing naloxone.
Nielsen et al. [58] (2016)	To explore the level of support for overdose prevention, the barriers and facilitators to naloxone supply, and the level of knowledge about naloxone administration among Australian pharmacists.	An online survey was distributed to community pharmacists across Australia, with 595 responses.	Community pharmacists surveyed state there may be insufficient time to properly train clients on naloxone administration.
Nguyen et al. [59] (2020)	To identify components leading to successful naloxone distribution from pharmacies, to evaluate the perceptions held by pharmacy staff regarding those who receive naloxone, and to assess relationships between these perceptions and the distribution of naloxone from pharmacies.	Semi-structured interviews were conducted with 14 pharmacists and pharmacy technicians across pharmacies in San Francisco.	The cost of naloxone was identified as a barrier to access. Establishing the community pharmacy as an encouraging, nonjudgmental environment was discussed as a means of addressing stigma.
Olsen et al. [60] (2019)	To examine the attitudes and experiences of Australian pharmacists regarding dispensing naloxone without patients needing a prescription.	Semi-structured interviews were conducted with 37 community pharmacists.	System-level barriers to dispensing naloxone identified include lack of education and training for dispensing naloxone, supply issues, lack of notification of changes in drug scheduling. Other barriers identified include stigma towards drug use, pharmacists unwilling to take extra time to educate staff and patients, the belief that dispensing naloxone would attract undesirable clientele.
Punches et al. [61] (2020)	To assess perceptions held by emergency nurses regarding take-home naloxone.	In-depth interviews were completed with 17 participants,	Some participants believed that naloxone enabled and condoned risky opioid use.
Richert [62] (2015)	To assess how people who use heroin understand the overdoses of others and make sense of their own and others’ responses to witnessing an overdose.	In-depth interviews were conducted with 35 Swedish heroin users.	Respondents believe that naloxone administrators may be reluctant to contact emergency services due to legal repercussions.
Rudolph et al. [63] (2018)	To identify barriers and areas for additional training in the dispensing of naloxone in community pharmacy settings.	An internet-based questionnaire was distributed to community pharmacists in North Carolina, with 423 respondents.	Community pharmacists remarked that naloxone administration may lead to continued or riskier opioid use and that the costs of naloxone are prohibitive to clients.
Samuels et al. [64] (2016)	To assess perceptions of opioid harm reduction interventions and willingness to perform them among ED physicians.	A web-based survey was distributed to ED physicians, of which there were 200 respondents.	Surveyed pharmacists believed that there may be insufficient time to properly train clients on naloxone administration.
Schneider et al. [65] (2021)	To assess knowledge of locations of accessible naloxone and perceived ease of accessing naloxone among suburban people who use opioids.	Computer-assisted self-interviews were conducted with 171 respondents.	Having knowledge of multiple sites of naloxone distribution and previous access increased ease of access among participants. Distance from harm reduction programs may make access more difficult.
Sisson et al. [66] (2019)	To better understand barriers that may prevent the use of naloxone programs operating out of pharmacies in Alabama.	Telephone surveys were conducted with 222 pharmacists across rural and urban areas in Birmingham, Alabama.	Perceived barriers to uptake of naloxone service include the high cost to patients and pharmacies and the belief that providing naloxone will lead to riskier opioid use.
Tewell et al. [67] (2018)	To describe the establishment of a pharmacist-led clinic where individuals in need of naloxone can be identified, provided education about risks and treatment of opioid overdose, and given naloxone.	During the pilot implementation, discussions were had with patients about their experiences.	Perceived barriers to naloxone access include lack of access to transportation, denial of the need to participate in the program, and stigma toward opioid use.
Thakur et al. [68] (2020)	To examine the roles of pharmacists, barriers, and pharmacist training, for dispensing naloxone from pharmacies.	A systematic review was conducted.	The following perceived barriers to dispensing naloxone identified: lack of training on how to identify and educate patients at risk of opioid overdose, prohibitive cost to patients, belief that dispensing naloxone encouraged opioid abuse, and belief that dispensing naloxone attracted an undesirable clientele.
Thompson et al. [69] (2018)	To examine Ohio pharmacists’ knowledge of naloxone, perceived barriers to naloxone dispensing, as well as confidence, comfort, and experience dispensing naloxone.	E-mail questionnaires were distributed to Ohio pharmacists, with 170 responses	Respondents surveyed believe that naloxone administration may lead to continued or riskier opioid use, that distributing naloxone through their pharmacy may invite clientele that would negatively affect their business, and that naloxone administration does not lead to compensatory or riskier opioid use.
Tobin et al. [70] (2009)	To evaluate the Staying Alive (SA) OEND program in Baltimore, Maryland.	Training for the SA program was given with pretest and posttest surveys, which were completed by 85 trainees.	Among trainees, there was fear of legal repercussions for calling emergency services when responding to an opioid overdose.
Tofighi et al. [71] (2021)	To assess attitudes and experiences of community pharmacists related to provisioning naloxone in non-urban areas of New York State.	Semi-structured surveys were provided to 60 community pharmacists.	A minority of participants believed that naloxone provision increased opioid use.
Young et al. [72] (2019)	To better understand the perceptions of those involved with the rapid increase of naloxone kit production and distribution as part of the British Columbia Take Home Naloxone (BCTHN) program, in terms of the challenges, facilitators, and successes they experienced.	Focus groups and key informant interviews were conducted with 18 stakeholders from a variety of groups involved in the ramp-up of the BCTHN program.	Facilitators identified include increasing the supply of naloxone, changing drug scheduling of naloxone, and addressing stigma toward drug use
Zaller et al. [73] (2013)	To examine the feasibility of implementing pharmacy-based THN programs in Rhode Island.	In-depth interviews were conducted with 21 PWID and 21 pharmacy staff.	Respondents perceived both THN clients and pharmacists as not being willing to participate in the program. As well, respondents believed the costs of naloxone are potentially prohibitive to clients.

Results and analysis

Key Findings of the Review

Four major themes emerged from the analysis of the literature review that examined the barriers and facilitators to accessing community naloxone programs by people who use opioids: (1) Stigma as a barrier to access; (2) Lack of a wide range of stakeholder perspectives; (3) Need for a comprehensive understanding of factors affecting equitable access to naloxone programs; (4) Facilitators to increase access of community naloxone programs.

Stigma as a Barrier to Access

Stigma was found to be a potential barrier to participation in naloxone programs [28,33,36,41,46,50-51,54-55,67,72,74]. When people who use opioids had not previously experienced an overdose, there was often denial that they would ever be at risk of overdosing and requiring naloxone treatment [67]. Oftentimes, people who use opioids were reluctant to utilize the program or to follow the instructions to call for an ambulance after using naloxone for the fear of legal repercussions [31,38-39,47,62,70,74]. Relatedly, people who use opioids may face stigma from first responders, including law enforcement, as well as from the general community [37]. Lack of information surrounding the legal liability of those administrating naloxone to someone who has overdosed was also found to be a major barrier to utilization [24,33,43]. Some studies highlighted the need for public education by healthcare providers to raise awareness of misinformation related to opioids and naloxone with the aim of reducing stigma and discrimination [28,40,54,67,74-75].

In addition, healthcare providers perceived that clients limited their interactions due to their fear of discrimination when accessing services [46,50,73]. Reviewing this literature revealed that there is a misconception among healthcare providers that distributing naloxone may promote continued or riskier opioid use [30,32-33,42,45,49,51,54,60-61,63,66,68-69,71]. Alternatively, healthcare providers perceived that naloxone distribution could pose a safety concern to the general public as a result of the potential aggressive behaviors associated with the naloxone recipient’s withdrawal side effects [24,33]. Additional sources of stigma perceived by healthcare providers include the misconception that naloxone programs will bring “undesirable” clientele to the local pharmacy and may bring undesirable effects to the community [30,42,50,60,68-69]. Community pharmacists specifically have been cited as gatekeepers to naloxone access, acting as a facilitator or barrier to receiving naloxone kits based on their knowledge of naloxone as well as the perceptions of individuals seeking the medication [44]. There needs to be an increased emphasis on educational interventions about naloxone programs to challenge these societal beliefs, as well as the attitudes and misconceptions of healthcare providers, which may contribute to the marginalization or victim-blaming of naloxone program users.

Lack of a Wide Range of Stakeholder Perspectives

Increasingly, users of naloxone distribution programs are involved as key stakeholders to share their lived experiences and perspectives in research studies [15-16,28,38,55]. However, many of the naloxone program studies conducted thus far focused primarily on either pharmacists [42,63] or physicians [23,43] while other important stakeholders, such as nurses, were rarely included [61]. In most cases, major emphasis was placed on the interaction between pharmacists and clients to understand their perspectives as providers and users of naloxone programs [46,67]. Besides the emphasis on physicians, pharmacists, and program users, no additional stakeholder group from the naloxone distribution programs were involved as study participants in these studies. These findings revealed the lack of perspectives from those who are involved in and actively contributed to the delivery of naloxone programs, including healthcare professionals such as nurses, program administrators, decision-makers, and policy-makers. Particularly, there is a need to examine a wider variety of perspectives from the diverse groups of key stakeholders who can potentially provide greater depth and breadth to the understanding of underlying barriers and facilitators to the equitable access of naloxone programs by marginalized and socially disadvantaged populations.

Barriers to Ease of Access for Vulnerable Populations

Our review revealed the lack of literature that focused on inequity associated with opioid-related health outcomes or strategies to minimize barriers and promote access by the vulnerable groups who are disproportionately experiencing opiate-related harms, including individuals with disabilities and without accessible means of transportation [67], as well as low-income individuals, particularly those without stable housing [36,74]. These vulnerable groups reported barriers related to the varying number of nearby community pharmacies with naloxone distribution programs in different areas [34,65] or the loss of naloxone kits during transient housing [74]. A study conducted by Mitchell et al. (2017) underscored the need for the placement of naloxone kits in common spaces of low-income housing in order to facilitate better access to naloxone by the marginalized populations [16]. Not being aware of naloxone programs, not having previously accessed naloxone, and not having knowledge of multiple sources of naloxone were found to negatively impact access [65]. Furthermore, our review revealed various studies of naloxone programs in North America, including North Carolina [63,67], Massachusetts, and Rhode Island in the United States [46], as well as Alberta [41] and British Columbia in Canada [16,74]. There is a need to expand our understanding of the challenges that are specific to different types of naloxone programs. For instance, barriers to naloxone program access may be limited by the need for clients to obtain a prescription from a physician or pharmacist in order to receive naloxone in certain jurisdictions [67]. Meanwhile, no prescription is needed to receive naloxone kits from distribution sites in all provinces in Canada (CRISM, 2019). An additional barrier to access identified in many jurisdictions involved the cost of naloxone to the recipient [24,30-31,33,48,50,56,59,63,66-68,73]. On the other hand, naloxone kits acquired through the THN programs in Canada are available at no cost, a method found to increase access to programs [52]. Future research is needed to explore the perspectives of key stakeholders involved in the naloxone distribution programs in different settings, which could help increase our understanding of the unique program strengths and challenges, as well as the implications of lessons learned in addressing the opioid crisis as it exists in Canadian, American, and global contexts.

Need for a Comprehensive Understanding of Factors Affecting Equitable Access to Diverse, Extant Naloxone Programs

A review of the existing literature revealed a major emphasis on the explorations of barriers related to the implementation of naloxone distribution programs while limited literature focused on the examination of barriers and facilitators to program access. For example, a study by Lacroix et al. (2018) focused on the implementation of a take-home naloxone program in emergency departments (EDs) across Canada [23]. The findings indicated that barriers to implementation included the lack of allied health support for client education; lack of time devoted to educating patients; and inadequate follow-up with the recipient of the naloxone kit. Lack of knowledge about naloxone programs and discomfort in distributing naloxone kits were also seen from programs that were established in different jurisdictions [33,54,57]. A perceived lack of time for adequate client education and training was frequently mentioned as a barrier [23-24,30,41-42,51,56,58,60,64,68]; this could be addressed by the adoption of mitigating strategies. Such strategies include determining and maintaining a constant supply of naloxone, as well as ensuring that all healthcare disciplines responsible for distributing naloxone have a high degree of knowledge of and comfort with naloxone use, in order to better equip the healthcare workforce in addressing the opioid crisis [76]. Specifically, these findings underscore the research gap that exists in the examination of health inequities related to naloxone program access by vulnerable groups of opioid users who are marginalized and socially disadvantaged such as those experiencing poverty, indigenous populations, immigrants, and refugees.

Facilitators to Increase Access of Community Naloxone Programs

The literature discussed many different facilitators, both potential and extant, to increase the accessibility of naloxone. Often mentioned as a facilitator to access was providing naloxone at no cost or as low a cost as possible [52]. One suggestion of a potential facilitator to access included working to create a non-judgmental space where naloxone can be provided [59]. Other literature suggested providing naloxone by default to anyone receiving any other supplies from harm reduction programs [52].

Discussion

Our review revealed that stigma surrounding opioid use and interactions with healthcare professionals (i.e. nurses, pharmacists, and physicians) was the most commonly reported barrier to naloxone access. The stigma and misconception toward substance use are also reflected in the general Canadian and American populations with approximately 89.7% and 89.5%, respectively, responding that they would not wish to have “drug addicts” as neighbors to the World Values Survey taken between 2017 and 2020 [77]. In particular, the existing literature revealed a major emphasis on examining the perspectives of physicians and pharmacists while other important stakeholder groups, such as nurses, were rarely given the opportunity to contribute their perspectives about their role in addressing the health inequities that exist in naloxone program access.

The lessons learned from our review included the promotion of facilitators to program access, such as re-structuring the interactions between program users and service providers to allow for more indirect communication and interaction about the request for naloxone treatment to avoid the risk of exposing the program user as a person who uses drugs (PWUD), as a stigmatized group [46]. Another implication of our analysis highlighted the need for raising awareness from healthcare disciplines about the misconception of significant compensatory use of opioids after naloxone has been given to clients [69]. Raising awareness of evidence-based information related to opioid use, coupled with empathetic narratives, could potentially help reduce stigma and increase support for the utilization of THN programs [29,78]. Service providers in public health and community settings should adopt the advocacy approaches as recommended by Cohen (2010) to enact a social justice practice in naloxone programs, including equipping oneself with evidence-based information about naloxone use, as well as challenging societal prejudice that leads to health inequities and stigma toward individuals who use opioids [79].

This review underscored the importance to implement social justice-focused research to examine the development of future innovations in naloxone programs, practice, policy, and education aimed at promoting social justice and equity among the people who use opioids. Currently, the methodological approach to the understanding of naloxone programs focused primarily on quantitative studies with descriptive approaches to gain insights from program participants through expert opinions, as well as surveys or questionnaires among individuals with lived experiences [23]. The structure of surveys and questionnaires has a limited ability to capture the in-depth ideas, perspectives, and experiences of study phenomenon [80], which necessitate the need for utilizing qualitative methodology to gain an in-depth understanding of the lived experiences of naloxone program users, as well as gaining insights from stakeholders with different roles who contribute to the naloxone programs through practice, administration, education, and research [41]. For instance, social justice-focused research could adopt a qualitative design through the use of semi-structured interviews [55], focus groups [46], and community-based participatory research (CBPR) [16]. More specifically, methodological approaches, such as grounded theory [43]; qualitative descriptive approach [74]; interpretative phenomenological analysis (IPA) [55]; as well as the application of Health Belief Model and Harm Reduction Frameworks can guide the research study of naloxone programs [16]. The Harm Reduction Framework can be the appropriate conceptual underpinning for social justice-focused research about naloxone access because of its emphasis on reducing harm from substance use without the need for abstinence while creating a safe, nonjudgmental environment where harm reduction can take place [35,81-82]. Future research about naloxone programs should embrace the complementarity of mixed methods, using quantitative and qualitative studies to explore strategies that challenge the societal stigma, policies, or practices leading to the marginalization or victim-blaming of people who use opioids experiencing health disparities.

## Conclusions

Our review highlighted the importance of advocacy in addressing the health inequities that exist in naloxone distribution programs in Canadian, American, and global contexts. This advocacy involves engaging in social justice practice through evidence-based informed research about the facts of opioid use, challenging the stigma toward naloxone program users, as well as promoting program development and health policy to bring about equitable access to naloxone programs by the marginalized and socially disadvantaged populations. As a result of the high number of opioid-related deaths and inequity in opioid-related harms, social justice-focused research is needed to examine how to improve access to naloxone programs and find effective ways to reduce health disparities among program users influenced by the social determinants of health. This knowledge can be used to inform and develop future innovations in education, research, and health policy aimed at promoting social justice and equity for all naloxone program users.
